# The effects of first defoliation and previous management intensity on forage quality of a semi-natural species-rich grassland

**DOI:** 10.1371/journal.pone.0248804

**Published:** 2021-03-30

**Authors:** Klára Pavlů, Teowdroes Kassahun, Vilém V. Pavlů, Lenka Pavlů, Petr Blažek, Petr Homolka

**Affiliations:** 1 Department of Ecology, Faculty of Environmental Sciences, Czech University of Life Sciences, Praha, Czechia; 2 Department of Nutrition and Feeding of Farm Animals, Institute of Animal Science, Praha, Czechia; 3 Grassland Research Station Liberec, Department of Weeds and Vegetation of Agroecosystems, Crop Research Institute, Praha, Czechia; 4 Department of Botany, Faculty of Science, University of South Bohemia, České Budějovice, Czechia; 5 Institute of Entomology, Academy of Sciences of the Czech Republic, České Budějovice, Czechia; 6 Deparment of Microbiology, Nutrition and Dietetics, Faculty of Agrobiology, Food and Natural Resources, Czech University of Life Sciences, Praha, Czechia; Government College University Faisalabad, PAKISTAN

## Abstract

Semi-natural grasslands occupy large parts of the European landscape but little information exists about seasonal variations in their nutritive value during the growing season. This paper presents results of novel data showing the effect of 13 years of previous contrasting management intensities on herbage nutritional value in relation to different dates of first defoliation (by grazing or haymaking). The treatments were: extensive management and intensive management from previous years (1998–2011). Both treatments were cut in June followed by intensive/extensive grazing for the rest of the grazing season (July–October). To evaluate forage quality in the first defoliation date, biomass sampling was performed in the year 2012 for 23 weeks from May to mid-October, and in 2013 for seven weeks from May to mid-June. Sampling was performed from plots that were not under management during the sampling year. Previous extensive management was associated with significantly reduced forage quality for in vitro organic matter digestibility (IVOMD), crude protein, neutral detergent fibre, acid detergent fibre and reduced divalent cations (Ca, Mg) and Na during the first seven weeks of the grazing season and the forage was suitable only for beef cattle. Due to low forage IVOMD, the forage is suitable only for cattle maintenance or for low quality hay when the start of grazing was postponed from seven weeks of vegetative growth to 13 weeks, regardless of the previous intensity. Herbage harvested after 13 weeks of the grazing season was of very low quality and was unsuitable as a forage for cattle when it was the only source of feed. Agri-environmental payments are necessary to help agricultural utilisation to maintain semi-natural grasslands by compensating for deterioration of forage quality, not only for the postponement of the first defoliation (either as cutting or grazing) after mid-June, but also when extensive management is required.

## Introduction

Permanent grasslands comprise about 35% of the total utilized agriculture area in the EU-28 countries of Europe [1, 2]. They provide not only forage for livestock, but also support other ecosystem services including carbon sequestration, and provision of landscapes and habitat [3]. Until the mid-twentieth century permanent grasslands were one of the most important feed sources for ruminant nutrition. Intensification of grassland managements (amelioration, reseeding with high productive mixtures, fertilization) and introduction of intensive milk production based on maize silage and concentrate mixtures, has resulted in semi-natural grasslands losing their main role of supplying feed for ruminants [4]. Nowadays, large areas of the semi-natural low-production grasslands in Europe that are characterised by rich floristic composition are managed under various types of agri-environmental schemes. These schemes frequently involve a reduction of management intensity and delaying the first cut or early season grazing in order to allow flowering of target species or to protect ground nesting birds. The result is the reduction of forage quality, especially digestibility of organic matter, in comparison with values from intensively managed grassland. In EU reduced forage quality is compensated by the different payment schemes to farmers that are under agri-environmental schemes [5].

Forage quality and biomass yield are the most important factors that affect decisions about the date of harvest of grassland. Achieving high forage quality together with high herbage production has been an important goal in grassland research in the context of intensive grassland management [6]. Therefore, there is much information available concerning the utilisation of high-production grasslands, particularly sown swards. On the other hand, there is considerably less information about forage quality and production of semi-natural species-rich grasslands, although such information is necessary for determination of appropriate management of grassland managed under agri-environmental measures [7]. Further, there have been few studies of changes in forage quality in relation to ageing of swards during the vegetation season [8–11]. Generally, fibre contents (acid detergent fibre (ADF) and neutral detergent fibre (NDF)) show a progressive increase but in vitro organic matter digestibility (IVOMD), nitrogen and phosphorus concentrations (’dilution effect’) generally decrease with ageing of the forage during the vegetation season [6, 11–13]. Forage in the early part of the growing season (or in new regrowth) usually has high digestibility values but low herbage yields; in contrast, with increasing maturity and net accumulation, biomass yields increase but there is also an increase in cell wall content and a decline in digestibility [6]. Therefore, for livestock farmers utilising semi-natural grassland, there are important questions concerning the most suitable time to start the grazing season or to apply the first cut, if grazed or mown herbage is to support the nutritional and mineral requirements of cattle. The suitability of the time of grazing or mowing is affected not only by herbage maturation but also by the type of vegetation, weather conditions and grassland management [14].

Where grassland is managed for conservation objectives within an agri-environmental programme, continual sampling of the grassland herbage during the vegetation season is necessary to determine the optimum range of dates for forage harvesting or grazing periods. However, very few such studies have been done [13]. Several studies have evaluated the forage quality of semi-natural low-production grasslands [10, 11, 13, 15], but these have not dealt with forage maturation during the vegetation season in relation to management intensity.

Semi-natural grasslands are an important part of European grasslands, and the *Arrhenaterion* alliance [16] with *Agrostis capillaris* and *Festuca rubra* dominance is one of the most widespread in Central Europe. However, not much is known about the nutritional properties of this grassland type in relation to the period of the vegetation season and management intensity. Within this context we aimed to answer the following questions: i) what is the impact of previous different grazing intensity types on dry matter standing biomass (DMSB), digestibility (IVOMD), concentrations of crude protein (CP), fibres (NDF, ADF), and macro-elements during the grazing season? ii) when is the appropriate period to introduce grazing or cutting of forage in order to meet cattle nutrition requirements?

## Materials and methods

### Study site

The study was conducted at ’Oldřichov Grazing Experiment’ located in the Jizerské hory Mountains in the northern part of the Czech Republic, in the village Oldřichov v Hájích, 10 km to the north of the city Liberec (50°50.34′N, 15°05.36′E; 420 m a.s.l.). This long-term experiment was established in 1998 [for details see 17]. We selected two treatments for this study where hay cutting (in June) was followed by aftermath intensive or extensive grazing.

The site has 30-year mean annual precipitation of 805 mm and a mean annual temperature of 7.2°C. Table 1 summarises the monthly rainfall and mean monthly temperature for the site (Liberec Meteorological Station). The bedrock is granite and medium deep brown soil (cambisol) with the following characteristics: pH (CaCl_2_) = 5.45, P = 64 mg kg^-1^, K = 95 mg kg^-1^ and Mg = 92 mg kg^-1^. There are about 24 vascular plant species *per* square metre, and the dominant species of the sward are *Agrostis capillaris*, *Festuca rubra* agg., *Trifolium repens*, and *Taraxacum officinale*. Since 1998 the mean cover of dominant vascular plant species was recorded by visual percentage estimation every year in spring before the first management application in all treatments of Oldřichov Grazing Experiment [for details see 17]. Table 2 shows this information for the years 1998 (base line), 2003, 2008, 2012 and 2013. The experimental area has been continuously stocked by young heifers (initial live weights of 150 to 250 kg), since 1998 from June (after cut) until mid or late October, however, the first week of May is the common period for starting the grazing season in this region. In the years 2002–2015 the mean total dry matter biomass production in the study area under intensive and extensive grazing ranged from 2.4 to 5.0 t ha^-1^ and from 2.3 to 4.7 t ha^-1^ respectively [18].

**Table 1:** Monthly precipitation (mm) and mean monthly temperature (^o^C) recorded in the years 2012 and 2013.

	Precipitation (mm)			Temperature (^o^C)		
Month/Year	2012	2013	1998–2013	2012	2013	1998–2013
January	134.9	99.2	72.8	-0.6	-2.3	-1.3
February	78.7	53.2	60.2	-5.4	-1.7	-0.5
March	34.6	35.8	63.6	4.8	-1.5	2.7
April	39.3	39.5	40.4	8.2	7.8	8.5
May	37.0	133.2	74.5	14.3	12	13.1
June	64.1	201.9	85.0	15.9	15.5	15.9
July	151.1	125.6	116.9	17.7	18.6	17.6
August	139.4	64.6	113.2	17.2	17.2	17.0
September	35.7	94.7	63.8	13.1	11.6	12.9
October	33.4	57.1	58.9	7.5	10.1	8.4
November	75.0	65.9	64.0	5.3	4.3	3.9
December	48.7	40.1	64.6	-0.9	2.4	-0.4
**Total Sum/Mean**	**871.9**	**1010.8**	**877.8**	**8.1**	**7.8**	**8.1**

Values are compared with the 16-year mean 1998–2013 (Liberec meteorological station).

**Table 2:** Mean botanical composition (%) of the most abundant vascular plant species.

Treatment	EG					IG				
Species/Year	1998	2003	2008	2012	2013	1998	2003	2008	2012	2013
*Aegopodium podagraria*	14	4	14	8	9	16	0	0	0	0
*Agrostis capillaris*	0	9	7	11	12	0	16	12	21	21
*Alchemilla* sp.	10	8	7	8	9	5	2	2	2	2
*Alopecurus pratensis*	28	3	4	8	9	22	3	4	1	1
*Festuca rubra* agg.	8	8	10	13	20	22	11	13	15	15
*Galium album*	15	8	10	5	5	6	0	1	1	0
*Hypericium maculatum*	1	2	5	7	9	5	0	0	0	0
*Poa trivialis*	2	3	6	3	3	2	3	14	16	18
*Ranunculus repens*	3	1	1	1	1	2	5	1	2	3
*Rumex acetosa*	1	3	5	3	2	2	1	3	4	4
*Taraxacum* spp.	2	26	14	13	12	2	22	29	22	32
*Trifolium repens*	0	13	3	1	1	0	33	24	18	9
*Veronica chamaedrys*	13	3	3	3	4	4	1	2	4	7
*Veronica serpyllifolia*	0	0	0	0	0	0	1	0	1	0

Numbers represent mean for the years 1998, 2003, 2008, 2012 and 2013 under extensive (EG) and intensive (IG) treatment.

### Experimental design

The experiment was established in two randomised blocks in the year 1998. Herbage sampling from two contrasting treatments were chosen: i) cutting in June followed by extensive grazing (EG) for the rest of the growing season, in which the stocking rate was adjusted to achieve a mean target sward surface height of more than 10 cm, and ii) cutting in June followed by intensive grazing (IG) for the rest of the growing season, in which the stocking rate was adjusted to achieve a mean target sward surface height of less than 5 cm throughout the grazing season. Both treatments were replicated twice in four plots. Each plot was approximately 0.35 ha.

### Data collection and laboratory analyses

The sampling area, a strip about 20 m x 4 m in each plot, was fenced with electric wire in 2012 and 2013 to protect the sward from grazing animals from the start of grazing season to the end of sampling period of each study year. Each year, the sampling area was situated on the opposite side of the plot. It allowed us to collect grassland biomass during maturation period which was affected by the different management intensity in the previous years (S1 Fig). Six randomly selected herbage biomass samples within 50 x 50 cm quadrats were cut by electric clippers once a week. To avoid repeated sampling from the same places, the sampling areas from where samples had been taken were marked with coloured sticks.

In 2012 the herbage biomass samples were collected from each paddock once a week from 2 May to 3 October (23 weeks of sampling x 2 treatments x 2 blocks x 6 samples; i.e. 552 samples in total) to determine forage quality throughout the whole grazing season. Concentrations of N, P, K, Na, Ca and Mg were determined from the 552 herbage samples collected. For analyses of IVOMD and fibres (ADF and NDF), samples were bulked to three *per* paddock. Since the main development on the forage quality was revealed during the first six weeks of sampling in the year 2012 (S2 and S3 Figs), we reduced the sampling from 23 weeks to seven weeks (early part of the grazing season) for the next grazing season in 2013.

In 2013 the herbage biomass samples were collected from each paddock once a week from 2 May to 13 June (7 weeks of sampling x 2 treatments x 2 blocks x 6 samples; i.e. 168 samples in total). Concentrations of N, P, K, Na, Ca and Mg were determined from the 168 herbage samples collected. For analyses of IVOMD and fibres (ADF, NDF) samples were bulked to three *per* paddock.

The fresh herbage biomass samples were weighed then oven dried (48 h at 60°C) to determine DMSB. Finally, samples were weighed and the dry herbage biomass was recalculated on a *per* ha basis, then milled and passed through a 1mm sieve. The concentration of N was determined by the Kjeldahl method [19] and then multiplied by 6.25 to obtain CP content. The concentrations of P, K, Na, Ca and Mg were determined by ICP-OES after digestion in *aqua regia* in an accredited laboratory of the Crop Research Institute in Chomutov. The NDF and ADF concentrations were specified according to the protocol described by [20] and [21] using the Ankom 200 Fiber Analyzer (Ankom Technology, Macedon, NY), analysed at the Institute of Animal Sciences in Prague. Digestibility (IVOMD) was determined by the Ankon Daisy incubator (ANKOM Technology) modification of enzymatic in vitro digestion method [22, 23] in the Institute of Animal Sciences in Prague.

The herbage samples chemically analysed for IVOMD, ADF and NDF collected in the year 2012 were further analysed by NIRS (FOSS NIRSystems 6500; NIRSystems, Inc., Silver Spring, USA) and calibration equations for IVOMD, ADF and NDF were calculated. The herbage samples collected in the year 2013 were analysed by the FOSS NIRSystems 6500 only.

The experimental land is not a part of any protected area and Crop Research Institute, Prague is the owner, therefore no specific permissions were required for this location. Further, we confirm that the field study did not involve any endangered or protected species.

### Data analysis

To obtain information about seasonal development of forage quality, data for the whole grazing season were collected in the year 2012 and are presented in the (S2 and S3 Figs). Based on the most important changes in forage quality in the year 2012, the first seven weeks period of sampling was chosen as a sampling period in the year 2013. Therefore, data from the first seven weeks of the grazing seasons of both 2012 and 2013 were statistically analysed.

A general linear model (GLM) with week (seven weeks as a continuous predictor) and treatment as fixed effects, with block and year as a random effects were used to analyse the effect of treatment, week and their interactions on DMSB, organic components (CP, IVOMD, ADF, NDF) and minerals (P, K, Ca, Mg, Na). Minerals data were log-transformed to meet GLM assumptions requirements. The effects were considered significant at the *P* < 0.05 level and Benjamini-Hochberg’s procedure was applied to control for false-discovery rate (FDR) [24]. All GLM analyses were performed in Statistica 13.1 [25].

## Results

### Dry matter standing biomass production

**Fig 1:**
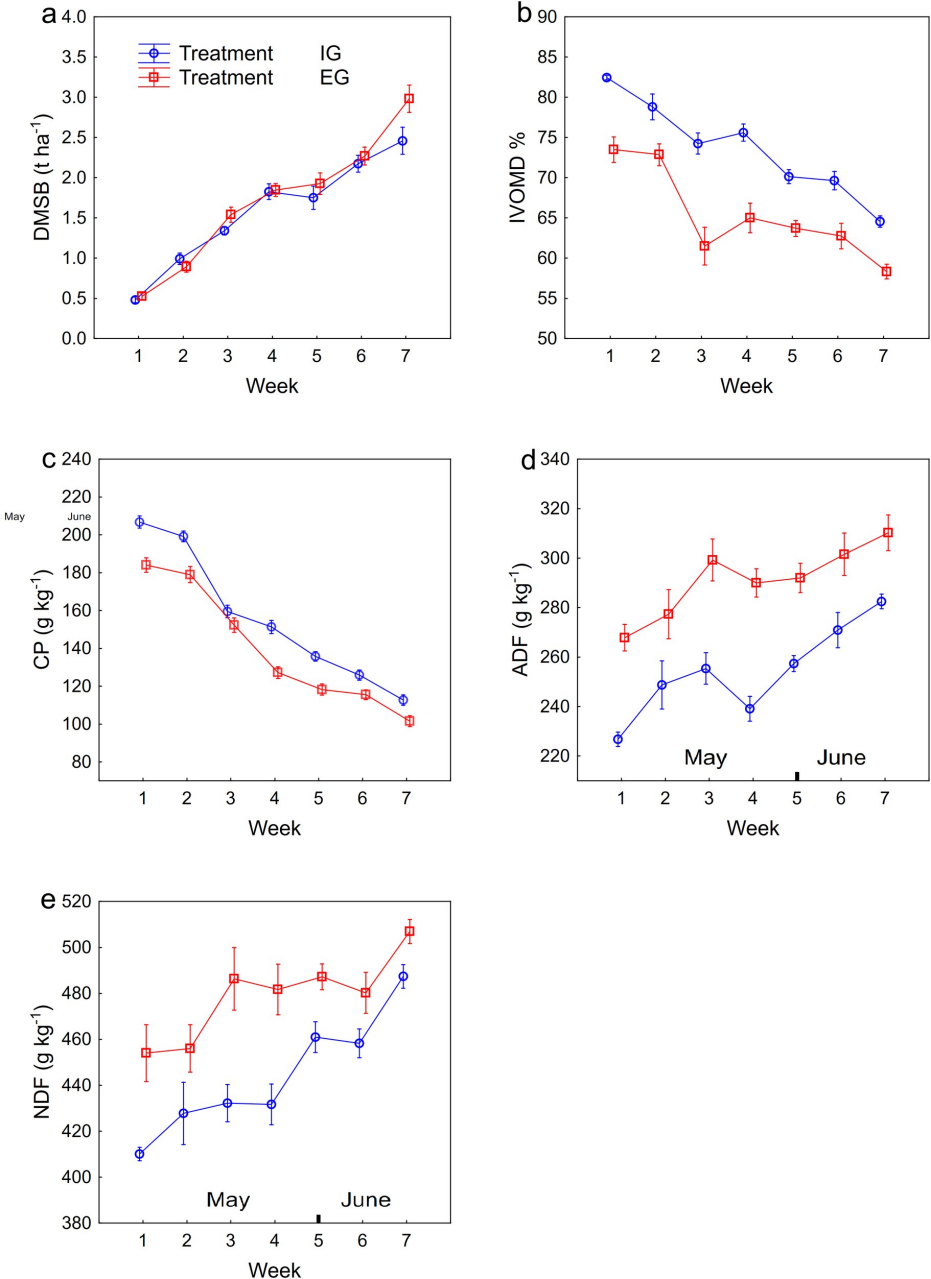
Mean dry matter standing biomass and organic components under extensive (EG) and intensive (IG) management. X-axis refers to the first seven weeks of grazing season in the years 2012 and 2013. Error bars represent standard error of the mean. For abbreviations see Table 3.

The DMSB was significantly influenced only by week (Table 3). In the early part of the grazing season DMSB had similar development till the sixth week in both treatments (Fig 1A); after that there was a tendency of divergence between the treatments with higher DMSB under the EG treatment. The highest mean value of DMSB in the EG treatment was recorded in the twentieth week (5.9 t ha^-1^) and in the IG treatment in the twenty-second week (5.3 t ha^-1^). From the eighteenth week to the end of the grazing season there was no development of DMSB under either treatment (S2a Fig).

**Table 3:** Results of GLM for DMSB, IVOMD, CP, ADF, NDF, P, K, Ca, Mg, Na, K/(Ca+Mg).

Characteristics	Effect	*Df*	*F-*ratio	*P-*value
DMSB	Treatment	326	0.36	0.549
	Week		638.24	**<0.001**
	Treatment x Week		3.21	0.074
**Organic components**				
IVOMD	Treatment	144	50.07	**<0.001**
	Week		217.53	**<0.001**
	Treatment x Week		3.96	0.048
CP	Treatment	309	33.29	**<0.001**
	Week		1156.61	**<0.001**
	Treatment x Week		4.10	0.044
ADF	Treatment	144	43.93	**<0.001**
	Week		93.73	**<0.001**
	Treatment x Week		2.41	0.123
NDF	Treatment	144	30.86	**<0.001**
	Week		87.41	**<0.001**
	Treatment x Week		5.36	**0.022**
**Minerals**				
P	Treatment	309	5.72	**0.017**
	Week		214.39	**<0.001**
	Treatment x Week		0.50	0.481
K	Treatment	309	0.02	0.884
	Week		61.71	**<0.001**
	Treatment x Week		0.04	0.845
Ca	Treatment	309	36.39	**<0.001**
	Week		7.56	**0.006**
	Treatment x Week		7.46	**0.007**
Mg	Treatment	309	60.57	**<0.001**
	Week		8.92	**0.003**
	Treatment x Week		8.75	**0.003**
Na	Treatment	309	32.95	**<0.001**
	Week		1.50	0.221
	Treatment x Week		5.34	**0.021**
K/(Ca+Mg)	Treatment	309	13.62	**<0.001**
	Week		55.88	**<0.001**
	Treatment x Week		3.06	0.081

Abbreviations: GLM—general linear model, DMSB—dry matter standing biomass, IVOMD—in vitro organic matter digestibility, CP—crude protein, ADF—acid detergent fiber, NDF—neutral detergent fiber. *Df* represents degrees of freedom, *F* represents the value derived from *F* statistics in GLM and *P* represents the resulting probability value. Results are summarized by denominator degrees of freedom *Df* (numerator *Df* was 1 in all tests). Significant results (after table-wise Benjamini-Hochberg’s FDR correction) are highlighted in bold.

### Organic components

The concentrations of IVOMD, CP, ADF and NDF were significantly affected by treatment and week. The concentration of NDF was significantly also influenced by treatment x week interaction (Table 3). During the early part of the grazing season a sharp decline in IVOMD was recorded in both treatments (Fig 1B). The mean values of IVOMD were significantly higher in the IG than in the EG treatment, and ranged from 64.5 to 82.5% in the IG treatment and from 58.3 to 73.5% in the EG treatment. From the eighth week till the end of the grazing season a moderate decline was recorded with the mean values in the range 43–55% in both treatments (S2b Fig).

Concentrations of CP and fibres (ADF, NDF) showed opposite development trends over the whole period of the grazing season (Fig 1C–1E; S2C, S2D and S2E Fig). In the early part of the grazing season CP concentration was significantly higher in the IG treatment than in the EG treatment, and mean values ranged from 101.5 to 184.0 g kg^-1^ for the EG treatment and from 112.6 to 206.8 g kg^-1^ for the IG treatment (Fig 1C). In the eighth week the mean values of CP concentration were about 100 g kg^-1^ in both treatments and they oscillated around this value till the end of the grazing season (S2c Fig). Fibre concentrations (ADF, NDF) were higher in the EG treatment in comparison with the IG treatment during the early part of grazing season. For ADF concentration the mean values ranged from 226.8 to 282.5 g kg^-1^ for the IG treatment and from 267.8 to 310.2 g kg^-1^ for the EG treatment. For NDF concentration the mean values ranged from 410.1 to 487.4 g kg^-1^ for the IG treatment and from 454.0 to 506.1 g kg^-1^ for the EG treatment in this period (Fig 1D and 1E). After the seventh week ADF and NDF concentrations were higher than 300 and 500 g kg^-1^, in both treatments respectively, (S2d and S2e Fig) though with no significant trend.

### Mineral nutrients

The concentrations of Mg and Ca were significantly influenced by treatment, week and interaction of week x treatment. The concentration of P and the K/(Ca +Mg) ratio were both significantly influenced by treatment and week. Concentration of Na was significantly influenced by treatment and interaction of treatment x week, and concentration of K was significantly influenced only by week (Table 3).

**Fig 2:**
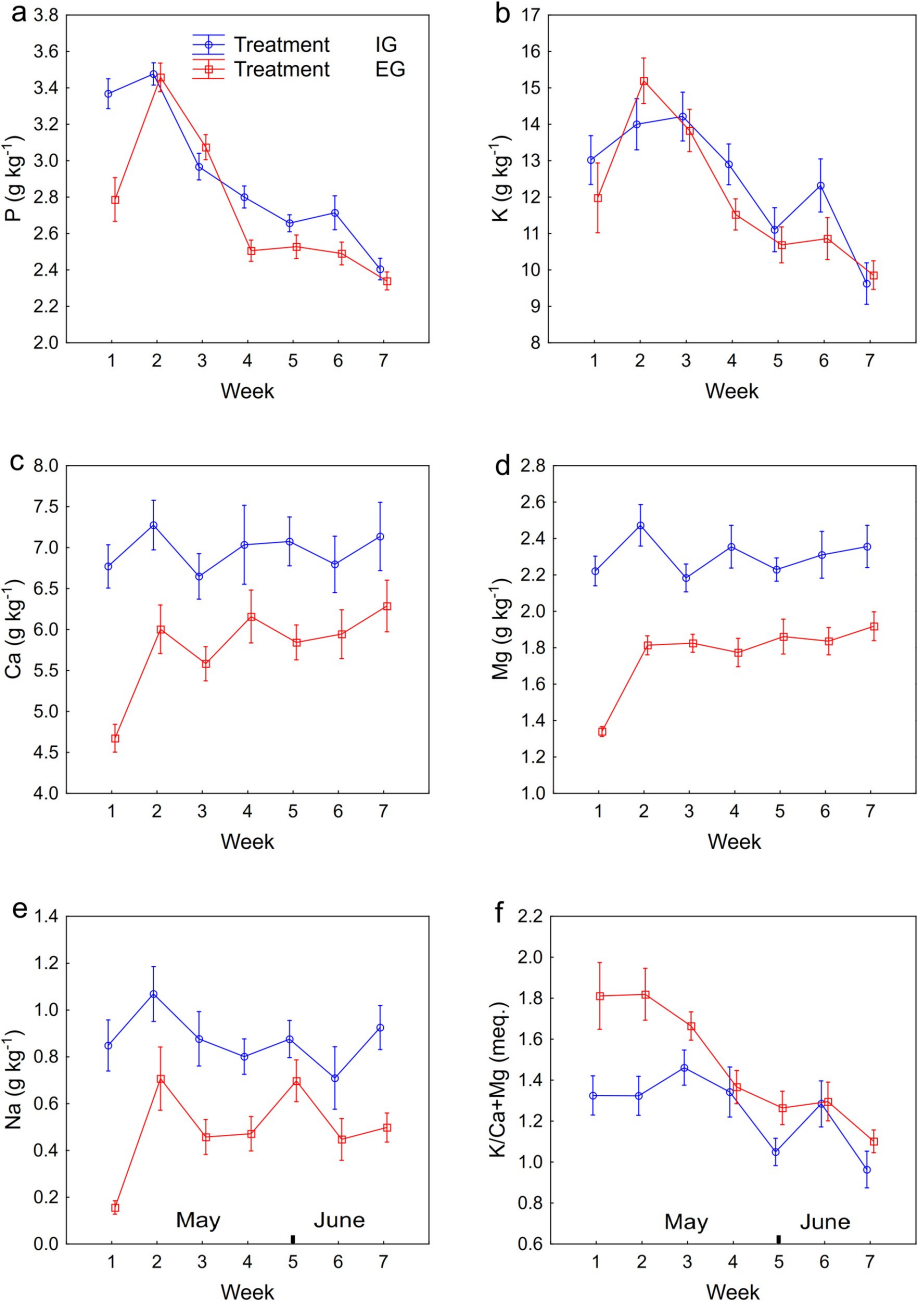
Mean concentration of minerals and K/(Ca+Mg) ratio under extensive (EG) and intensive (IG) management. X-axis refers to the first seven weeks of grazing season in the years 2012 and 2013. Error bars represent standard error of the mean.

The sharp decrease of P concentration in the herbage was recorded from the second to the seventh week for both treatments (Fig 2A) with the highest mean values of 3.5 g kg^-1^ in the second week in both treatments. From the eighth week the mean values were maintained at almost the same level for both treatments and their range was approximately between 1.9 to 2.5 g kg^-1^ till the end of the grazing season (S3a Fig).

In the early part of the grazing season the K concentration reached it highest peak in the second week under EG treatment and in the third week under IG treatment. There was then a decline in K concentration up to the seventh week in the both treatments with mean values ranging from 14.2 down to 9.6 g kg^-1^ in the IG treatment and from 15.2 to 9.9 g kg^-1^ in the EG treatment (Fig 2B). This declining trend was maintained for the rest of the grazing season (S3b Fig) in both treatments, with mean values ranging from 12.3 down to 8.3 g kg^-1^.

Concentrations of both cations Ca and Mg in the herbage were significantly higher in the IG than in the EG treatment in the early part of the grazing season (Fig 2C and 2D); nevertheless, no developmental trend was recorded in any treatment during this period. The mean values of Ca concentration in the herbage ranged from 4.7 to 6.3 g kg^-1^ for the EG treatment and from 6.7 to 7.3 g kg^-1^ for the IG treatment. The mean values of Mg concentration in the herbage ranged from 1.3 to 1.9 g kg^-1^ for the EG treatment and from 2.2 to 2.5 g kg^-1^ for the IG treatment in this period. From the ninth week onwards the herbage Ca concentration in the EG treatment tended to be higher than in the IG treatment, whereas Mg concentration was similar in both treatments for the remainder of the season (S3c and S3d Fig).

In the early part of grazing season Na concentration in the herbage was significantly higher in the IG than in the EG treatment; the mean values ranged from 0.2 to 0.7 g kg^-1^ for the EG treatment and from 0.7 to 1.1 g kg^-1^ for the IG treatment (Fig 2E). The concentration of Na in the herbage decreased during the whole of the grazing season in both treatments (S3e Fig).

In the early part of the grazing season the K/(Ca+Mg) ratio (meq.) showed a slow decline in both treatments and this ratio was significantly higher in the EG than in the IG treatment (Fig 2F). The mean values of the K/(Ca+Mg) ratio ranged from 1.0 to 1.5 for the IG treatment and from 1.1 to 1.8 for the EG treatment in this period. From the eighth week throughout the rest of the grazing season the mean values for the K/(Ca+Mg) ratio were predominantly higher in the IG than in the EG treatment (S3f); however, no development was observed in this period.

## Discussion

The timing of grazing activities and the grazing intensity are generally considered to be the key factors that affect both the quality and quantity of pasture forage [13, 15, 26, 27]. The stage of maturity of harvested herbage is affected by the date of harvesting and this greatly influences the overall forage quality, because of the increasing proportion of cell wall components during the growth of most grassland species [8, 11, 28].

During the early part of the grazing season rapid changes in forage quality and DMSB were found in our experiment. These occurred in both management intensities; nevertheless, the previous grazing intensity had a significant effect on value of many qualitative components of forage in this period. Of particular note was that parameters of forage quality in the EG treatment in the first week of the grazing season were negatively affected by the presence of overwintered herbage from the previous vegetation season.

### Dry matter standing biomass production

The DMSB development reflected typical biomass growth at the study site [18] and it was not affected by treatment during the early part of the grazing season. From the seventh week the value of DMSB started to increase under the EG treatment, although total biomass production was higher under the IG treatment in the plots that previously had been defoliated regularly [18]. It seems that the taller vegetation that developed under extensive management could provide higher DMSB than the short vegetation under the IG treatment [17].

### Organic components

Values of IVOMD and CP concentrations showed similar patterns over the course of the grazing season. In both treatments there was a sharp decline from the early part of the grazing season, as young forage in vegetative state has higher digestibility values and contains higher concentrations of N compared with more mature forage [13, 29, 30]. A gradual decrease of IVOMD as the sward herbage increases in maturity is usually linked to increasing accumulation of structural carbohydrates and lignification [6, 31] and this is also associated with a reduction in plant N content and therefore of CP. The optimal value of IVOMD required in forage for dairy cows is higher than 67% [6] but for beef cattle a lower threshold of at least 60% may be assumed [32]. A maintenance value of IVOMD in forage for cattle is around 50% [33].

In our experiment the optimum level of IVOMD required in forage for dairy cows was fulfilled during the first six weeks of the grazing season in the IG treatment but only during the first two weeks in the EG treatment. It means that the digestibility of forage is affected not only by the intensity of grazing during the recording period, as also shown in several studies previously [8, 34–37], but also that the grazing intensity applied during previous years can play an important additional role. In both the EG and IG treatments the value of IVOMD was suitable for feeding beef cattle during the whole early part of the grazing season, as beef cattle do not require forage to be of the high digestibility as that required by dairy cows [32]. In the period from the seventh week to the end of the grazing season 2012 the value of IVOMD seemed not to be affected by the previous grazing intensity, and maintenance values of IVOMD for feeding cattle were sufficient until the 13th week of the grazing season under both treatments. Similar IVOMD development is typical for upland European grasslands [e.g. 13, 38]. However, the herbage harvested after 13 weeks in the year 2012 was of very low quality and was not usable as the only source for feed for cattle, although such herbage may be used for combustion [11].

Higher proportion of legumes or *Taraxacum* species in the sward of the IG treatment could contribute to higher CP concentration in the herbage especially during the early part of the grazing season. These plant species usually have higher CP concentrations than occur in grasses [e.g. 39–41]. The concentrations of CP were appropriate for the requirements of dairy cows (>160 g kg ^-1^) [42] only for the first two weeks in both treatments. However, the low amounts of DMSB do not permit the economical utilisation of herbage biomass in this period. After a sharp decline during the first seven weeks the CP concentrations in the forage were about 100 g kg ^-1^ regardless of treatment, a level which still met the requirements for beef cattle (80 g kg ^-1^) [42].

In both the EG and IG treatments forage quality in terms of NDF concentration was not suitable for dairy cows at all, the acceptable threshold being about 300–400 g kg ^-1^ [43, 44]. The relatively high NDF concentration in the forage means that it is useable only for beef cattle [32]. Except for the first week in the IG treatment, the concentrations of ADF in forage of both treatments were so high as to be considered not acceptable for dairy cows, as recommended thresholds for dairy cows are about 190–240 g kg ^-1^ [43, 44]. After the first seven weeks of the vegetation season in the year 2012 both NDF and ADF concentrations in the herbage increased and remained suitable only as forage for beef cattle [32].

### Mineral nutrients

The concentrations of minerals in the herbage are mainly affected by the nutrient concentration in the soil [45], and also by phenophases and representation of individual agro-botanical groups in grassland during the vegetation season [10]. Other factors, such as shading intensity, soil moisture and pH, may also affect mineral concentrations in the herbage biomass [45]. During the grazing season a significant decline of P, K and Na concentrations occurred, most likely due to the ’dilution effect’ described by [12], in which during the maturation the herbage biomass increases whereas mineral concentration declines [10, 46]. Dairy cows have greater nutritional requirements for P, K, Ca, Mg and Na minerals than beef cattle and sheep, mainly due to the needs of lactation [30].

In both the EG and IG treatments dietary concentration of P in herbage met the requirements of productive animals (2.4–4.0 g kg^-1^, [30]) only during the first six weeks. After sharp decline in the first seven weeks of grazing season P concentration was relative stable in the rest of grazing season; nevertheless, they were mostly below recommended threshold [30].

Potassium was the only mineral that exceeded the recommended range for cattle nutrition (5–9 g kg^-1^, [30]) during almost the whole grazing season in both treatments. Especially in the spring, K concentration in the biomass was high, but during the course of the vegetation season it decreased gradually, a finding also described by [47]. The physiological requirements of K for animals tend to be significantly lower than is usually present in herbage [30, 48]. However, due to high Ca and Mg concentrations in the herbage in our experiment the grass tetany ratio K/(Ca+Mg) in meq. of 2.5 [49, 50] was never exceeded.

The concentration of Ca in the IG treatment in the early part of grazing season was sufficiently high to meet nutritional requirements for dairy cows (4–6.0 g kg^-1^, [30]). It was probably caused by higher proportions of legumes and *Taraxacum* species in the IG treatment as these species contain high concentrations of Ca [30, 48, 51–54]. In later periods the relative proportions of legumes and *Taraxacum* species decreased with increased growth of grasses (*Agrostis capillaris*, *Festuca rubra* agg., *Poa trivialis*), which have generally lower mineral concentrations than forbs [55]; together with the ’dilution effect’ this resulted in a decline in Ca concentration with maturation of the sward. In this period Ca concentration in the IG treatment was suitable only for low productive milking cows (threshold 3.0 g kg^-1^) and beef cattle (threshold 2.9 g kg^-1^) [30].

In the EG treatment the concentration of Ca, with no trend, mostly met the requirements for dairy cows during the whole grazing season. Its value was lower than in the IG treatment in the early part of grazing season only. Further, in the EG treatment in the late part of grazing season several tall forbs (*Aegopodium podagraria*, *Galium mollugo* agg., *Hypericum maculatum*), which would likely have had higher concentrations of Ca than grasses [55], increased their proportion in the sward at the expense of the grasses (unpublished observation). Thus, higher Ca concentration in the herbage in the EG treatment than in the IG treatment in the late part of grazing season could be caused by seasonal development of plant species composition, as described also by [10].

The concentration of Mg in the herbage fulfilled the requirements for dairy cows (at least 2.0 g kg^-1^) only in the early part of the grazing season in the IG treatment. During the later period the herbage was mostly suitable only for beef cattle (1.6 g kg^-1^) in both treatments [30].

The requirements for Na by dairy cows (2.0 g kg^-1^) as well as beef cattle (1.0 g kg^-1^) usually exceed the Na concentration present in herbage [30]. In our experiment concentration of Na in the forage was not sufficient for the requirements of either dairy cows (2.0 g kg^-1^) or beef cattle (1.0 g kg^-1^) [30] in both treatments during the whole grazing season in the year 2012. In general, however, it is usually possible to deal with mineral imbalances by supplying livestock with free-choice mineral supplements [48, 56].

## Conclusion

The previous extensive management had a carry-over effect which significantly reduced the quality of organic components (IVOMD, ADF, NDF, CP), divalent cations (Ca, Mg) and Na in herbage of *Agrostis capillaris* and *Festuca rubra* dominated grassland during the first seven weeks of the spring grazing season. Due to the high concentration of fibres (ADF, NDF) the forage was suitable only for beef cattle even during the first seven weeks of the grazing season. Besides Na and K, the concentrations of other tested minerals were in the range recommended for cattle feeding and were also affected by species composition of the sward. Herbage mineral concentrations declined over the course of the sward maturation. When the beginning of grazing or hay-making was postponed from the 7th to 13th week of the grazing season the forage was sufficient only for cattle maintenance (based on IVOMD) in both extensive and intensive treatments. Herbage harvested after 13 weeks had very low quality and was not suitable for use as the only source for cattle feeding.

Thus agri-environmental payments are necessary to compensate for deterioration of forage quality if the utilisation of semi-natural grassland is restricted for environmental reasons, and this will apply not only for the postponing of the first defoliation (either as cutting or grazing) to after mid-June, but also when extensive management is required.

## Supporting information

S1 Fig The design of the experiment. (TIFF)

S2 Fig Mean dry matter standing biomass and organic components under extensive (EG) and intensive (IG) management. Axis X refers to the whole grazing season (23 weeks) in the year 2012. Error bars represent standard error of the mean. For abbreviations see Table 3.
(TIF)

S3 Fig Mean concentration of minerals and K/(Ca+Mg) ratio under extensive (EG) and intensive (IG) management. Axis X refers to the whole grazing season (23 weeks) in the year 2012. Error bars represent standard error of the mean.
(TIF)
