# Supplementary figures and images for: The effects of first defoliation and previous management intensity on forage quality of a semi-natural species-rich grassland
Source: PLoS One. 2021 Mar 30;16(3):e0248804. doi: 10.1371/journal.pone.0248804 (PMC8009352; doi:10.1371/journal.pone.0248804)

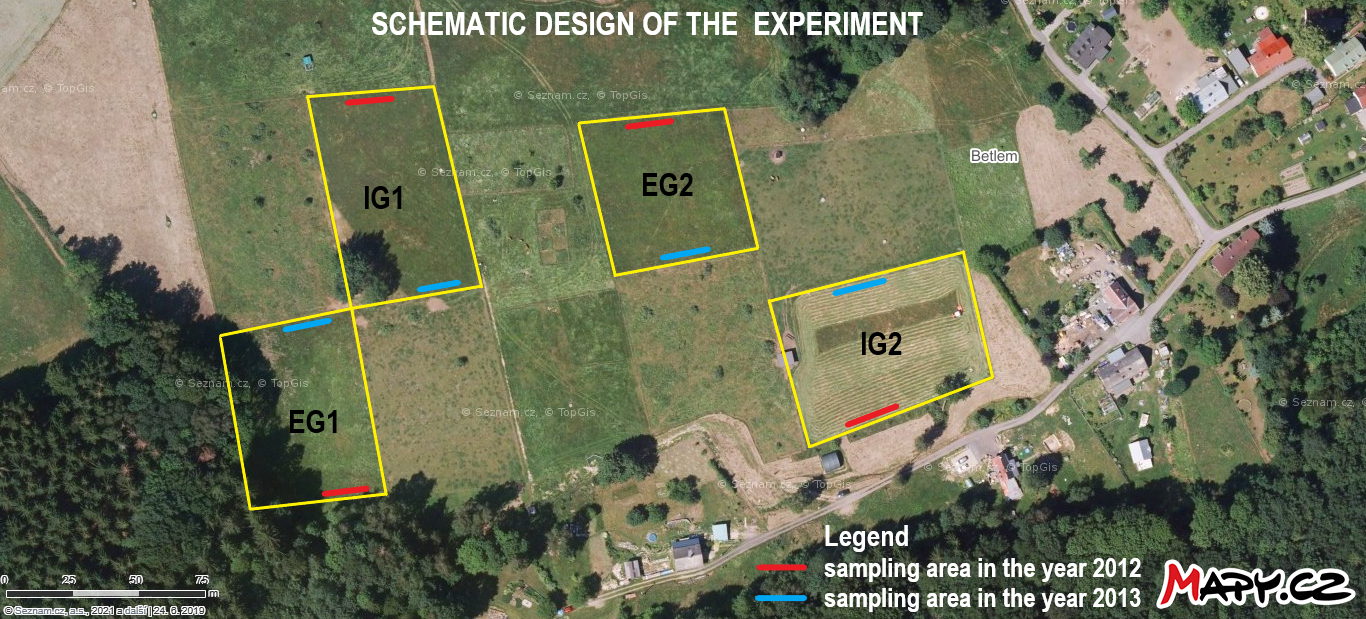

Supplement: S1 Fig — (TIFF) [file pone.0248804.s001.tiff]

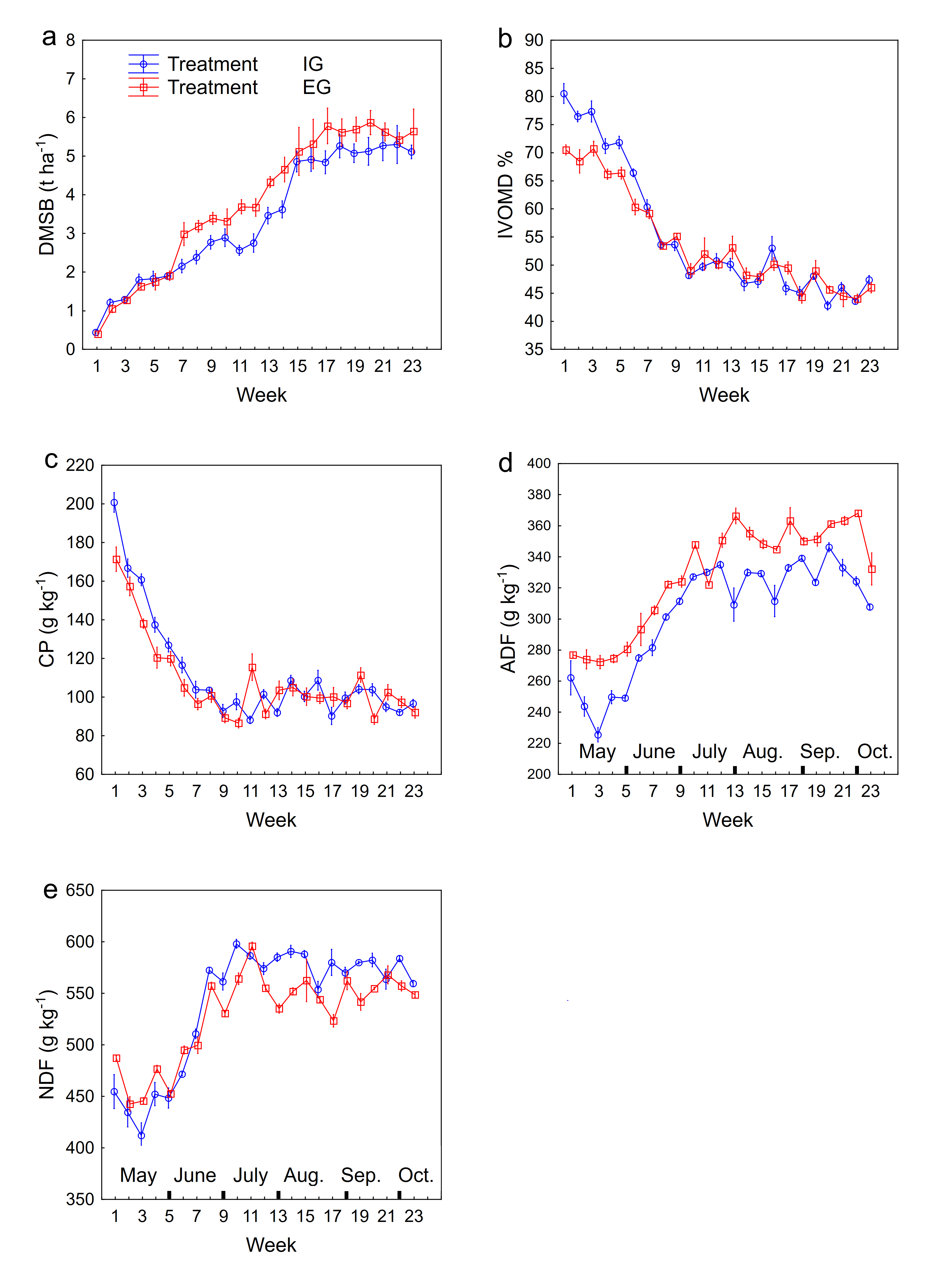

Supplement: S2 Fig — Axis X refers to the whole grazing season (23 weeks) in the year 2012. Error bars represent standard error of the mean. For abbreviations see Table 3. (TIF) [file pone.0248804.s002.tif]

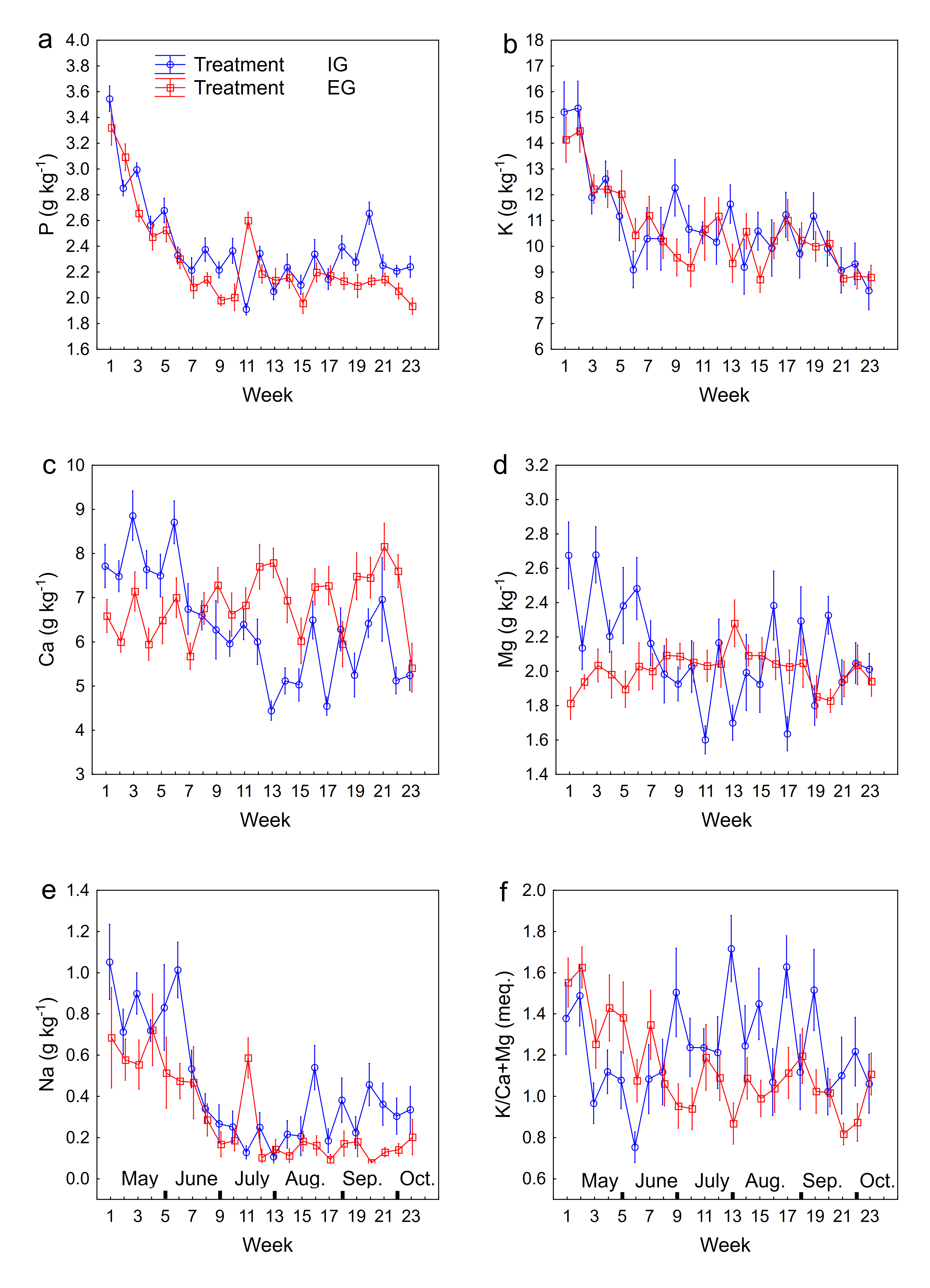

Supplement: S3 Fig — Axis X refers to the whole grazing season (23 weeks) in the year 2012. Error bars represent standard error of the mean. (TIF) [file pone.0248804.s003.tif]
